# Clinical experience with low-dose itraconazole in chronic idiopathic cough

**DOI:** 10.1186/1745-9974-9-1

**Published:** 2013-01-14

**Authors:** Haruhiko Ogawa, Masaki Fujimura, Yasuo Takeuchi, Koichi Makimura

**Affiliations:** 1 The Division of Pulmonary Medicine, Ishikawa-ken Saiseikai Kanazawa Hospital, Ni-13-6 Akatsuchi-machi, Kanazawa, 920-0353, Japan; 2 Respiratory Medicine, National Hospital Organization Nanao Hospital, Nano, Japan; 3 Division of Respiratory Medicine and Clinical Allergy, Fujita Health University, Toyoake, Japan; 4 Department of Molecular Biology and Gene Diagnosis, Institute of Medical, Mycology and Genome Research Center, Graduate School of Medical Science, Teikyo University Hachioji, Hachioji, Japan

**Keywords:** A sensation of mucus in the throat, *Bjerkandera adusta*, Chronic idiopathic cough, Fungus-associated chronic cough, The Japanese version of LCQ

## Abstract

**Background:**

The presence of basidiomycetous (BM) fungi in induced sputum is an important clinical finding in chronic idiopathic cough (CIC). However, the efficacy of anti-fungal therapy for CIC has not been evaluated.

**Methods:**

We selected 10 patients with CIC and carried out allergological examinations for *Bjerkandera adusta*, a BM fungus that has been shown to enhance cough severity. The efficacy of low-dose itraconazole (ITCZ) therapy (50 mg/day) for 14 days as an adjunctive therapy was estimated with use of Cough Visual Analog Scale (Cough VAS) and the Japanese version of the Leicester Cough Questionnaire (J-LCQ). We evaluated whether there was a recognizable clinical or allergological pattern that could predict the efficacy of ITCZ therapy in CIC patients.

**Results:**

Significant changes in Cough VAS and minimal important difference in domains of the J-LCQ were observed in 3 and 5 CIC patients, respectively. The Δ cough scale was correlated with changes in domains of the J-LCQ (total (r = –0.73, *P* < 0.05), psychological (r = –0.73, *P* < 0.05), and social (r = –0.71, *P* < 0.05), respectively. There were significant differences in the change in total score (*P* < 0.05) and in the domain of social (*P* < 0.05) and Δ cough scale (*P* < 0.05) between positive and negative results of immediate skin test for *B. adusta*. Positive results for improvement of cough-related laryngeal sensation which was represented as a sensation of mucus in the throat (SMIT) were observed in 6 patients in the BM colonization-positive group (85.7%) and none in the BM colonization-negative group (0%). There was a significant difference in the positive ratio for improvement of SMIT between the two groups.

**Conclusions:**

At present, it is not possible to conclude whether ITCZ therapy provides sufficient relief in CIC patients. However, this study suggested both the possible applicability of low-dose ITCZ therapy for treatment of CIC patients with regard to BM allergy and the necessity of development of a new assessment questionnaire for cough-related laryngeal sensations.

**Trial registration:**

UMIN-CTR (reference number R000005872; UMIN000004933).

## Background

Despite extensive diagnostic evaluation and numerous treatment guidelines [1-3], a subgroup of chronic cough patients in whom a diagnosis cannot be made even after thorough systematic investigation remain troubled by chronic idiopathic cough (CIC) [4,5], defined as an uncontrollable cough that is difficult to treat. Therefore, there is a great deal of research interest [6,7] regarding the identification of novel antitussive drugs [8-11] and the establishment of novel therapeutic strategies.

We recently encountered a cluster of patients with allergic fungal cough (AFC), which is intractable and is characterized by sensitization to *Bjerkandera adusta*[9], among a new clinical disease concept termed fungus-associated chronic cough (FACC) [10] with the following manifestations: (1) chronic cough; (2) the presence of environmental fungi, particularly basidiomycetous (BM) fungi, in the sputum; and (3) good clinical response to antifungal drugs. Thus antifungal drugs have provided a new treatment strategy for chronic intractable cough [11], included in unexplained chronic cough [12]; however, the efficacy of anti-fungal therapy for CIC has not been evaluated yet.

As the presence of BM fungi in induced sputum has been shown to be an important factor in distinguishing the clinical manifestations of CIC from those of non-CIC [13], we describe here our clinical experience in 10 CIC patients treated with low-dose itraconazole (ITCZ). We attempted to clarify the problems raised in the process of evaluating the clinical efficacy of low-dose ITCZ, especially with regard to both colonization of BM fungi and sensitization to *B. adusta*.

## Case presentation

### Methods

### Mycological study

#### Strains and DNA preparation

The sputum samples obtained from the patients with CIC patients were cultured on Sabouraud’s dextrose agar (SDA) containing chloramphenicol. The morphological features of the strains were observed with the slide culture method (30°C for 2~3 weeks). When the white colonies grew widely on SDA; the resulting colonies were moved onto CHROMagar® Candida spread with micafungin sodium (Funguard®, 30 μ/plate) [11].

### Allergological tests

#### Preparation of the antigenic solution

The antigens *Aspergillus, Alternaria, and Candida* were commercially available (Torii Pharmacy, Tokyo, Japan). One liter of Sabouraud dextrose broth in 3 L flasks was sterilized by autoclaving at 121°C for 20 min. Five milliliters of *B. adusta* (NBRC 4983) or *Schizophyllum commune* spore suspension (10^5^ spores/ml) in sterile physiological saline from 14 day-old SDA culture was used to inoculate the flask. The flasks were shaken at 150 rpm in a 25°C rotary shaker incubator. The supernatant was then dialyzed against 5 mM ammonium bicarbonate and lyophilized.

#### Intradermal skin test

An antigenic solution (polysaccharide) was injected intradermally with a tuberculin syringe (0.02 mL, 1 mg/mL) to assess the skin response to the solution. The results were judged to be positive when the longer axis of the flare exceeded 9 mm and/or ≥ 3 mm above negative control at 15 min (immediate phase), and 10 mm at 8 h (late phase) after the injection.

#### Serological test

House-dust, mite, *Aspergillus, Alternaria, Penicillium, Cladosporium, Candida Trichophyton;* Allergen-specific IgE antibodies were detected using a capsulated hydrophilic carrier polymer radioallergosorbent test fluoroenzyme immunoassay (Phadia, Uppsala, Sweden) at an external laboratory (SRL, Tokyo, Japan).

#### Lymphocyte stimulation test

The lymphocyte stimulation test (LST) [14] was performed using the antigenic solution with the Lymphoprep system. The results were considered positive when the magnitude of the response to *B.adusta* was beyond 200% in comparison to the controls using PHA.

### Diagnostic criteria of CIC

According to the Japanese Cough Research Society [1], Japanese Respiratory Society [2], and the American College of Chest Physicians evidence-based practice guidelines [3], the cause of chronic cough in each patient was diagnosed based on a questionnaire, blood examination findings, chest and sinus X-rays, induced-sputum examination, pulmonary function tests [15], test for cough reflex sensitivity to inhaled capsaicin [16], bronchial reversibility in response to bronchodilators, bronchial responsiveness to methacholine, and the efficacy of individual cause-specific treatments. The capsaicin cough threshold was defined as the lowest concentration of inhaled capsaicin eliciting five or more coughs (C5). Capsaicin reflex sensitivity was judged to be increased when capsaicin concentration eliciting five or more coughs (C5) was < 3.9 μM in males or < 0.98 μM in females [17]. Positive bronchial reversibility was defined as percentage increase in FEV1 > 12% and absolute increase in FEV1 > 200 mL. The non-specific bronchial responsiveness to methacholine was assessed according to the method described by Cockcroft et al. [18]. The results were expressed as the provocation concentration (mg/mL) required to cause a 20% or more fall from the baseline FEV1 (respiratory threshold of methacholine; RT-Meth).

The specific treatments given before the diagnosis of CIC was made were as follows: Suspected cough variant asthma [19] was treated in the first instance with β2-agonists (a combination of oral 40 μg/day clenbuterol and 200 μg salbutamol inhalation at bedtime and on demand). If this proved insufficient, treatment was stepped up according to the guidelines on the treatment of asthma. Suspected atopic cough [20], i.e., bronchodilator-resistant cough (eosinophilic tracheobronchitis with cough hypersensitivity), was treated with histamine H1 antagonists and inhaled corticosteroids (a combination of 10 mg/day cetrizine hydrochloride and 400–800 μg/day fluticasone propionate). Suspected sinobronchial syndrome [21] was treated with clarithromycin (oral 200 mg/day). Suspected gastro-oesophageal reflux [22] was treated with a high dose of proton-pomp inhibitors. The duration of each treatment was a minimum of 3 months.

### Assessment of treatment efficacy against cough symptoms

#### Cough Visual Analog Scale (Cough VAS)

A subset of subjects marked a 100-mm linear VAS to indicate severity of their cough from “no cough” to “worst cough.” The efficacy of the treatment was evaluated based on the change in the cough scale before and after treatment (Δ cough scale), and a change in Δ cough scale by more than 15 mm was taken to be significant [23].

#### Cough-related quality of life

The Leicester Cough Questionnaire (LCQ) [24] is a valid, reproducible, responsive, self-reported, cough-specific health status measure. The Japanese version of LCQ (J-LCQ) was adapted for Japanese conditions following a forward-backward translation procedure [25]. The LCQ total score ranges from 3 to 21 and from 1 to 7 for physical, psychological and social domains; a higher score indicates a better health-related quality of life. The mean (standard deviation) minimal important difference (MID) of the LCQ corresponding to a small change in the four Global Rating of Change Questionnaires (GRCQ) score was 1.3. The MIDs for domains were as follows: physical 0.2, social 0.2, and psychological 0.8 [26].

### Protocol

Ten patients diagnosed with CIC and treated for more than 2 years were selected for this study from 1 February to 30 April 2011. Allergological examinations were performed using an antigenic solution of *B. adusta*. The patients received a low dose of ITCZ (50 mg/day) for 14 days as adjunctive therapy. The efficacy of the drug was estimated with use of Cough VAS and the J-LCQ.

### IRB approval

This selected, open, single-arm, prospective trial was approved by the institutional review board of Saiseikai Kanazawa Hospital (reference number 2011007) and approval for the study was obtained from UMIN-CTR (reference number R000005872; UMIN000004933). A written informed consent was obtained from each patient prior to enrollment in the study.

### Statistical analysis

Variables are expressed as the mean (SD) unless otherwise stated. For comparison of multiple groups, analysis of variance (ANOVA) followed by Fisher’s protected least significant difference post hoc test was used for parametric data, when a significant difference was found. For nonparametric data, the Kruskal–Wallis test followed by the Mann–Whitney *U* test was applied instead. The *χ*^2^ test was used for categorical data. Analyses were performed using the SPSS statistical software package. In all analyses, *P* < 0.05 was taken to indicate statistical significance.

## Results

Ten patients with CIC had a median age of 65.0 (range, 47–71) years, and 70.0% were female. Chest and sinus radiographs were normal in all patients. Based on their clinical histories, the duration of cough ranged from 27 to 70 months. None of the patients complained of shortness of breath or wheezing and none had post-nasal drip or sinobronchial syndrome that could be responsible for the cough. The mean white blood cell count was 5365.0 ± 1423.6 (SD) μL with 1.8% ± 1.5% (SD) eosinophils in peripheral blood. The total serum IgE levels were found to be elevated in 2 patients and mean level was 132.8 ± 217.9 IU/mL (mean ± SEM). Mean lung functions were as follows: FVC 114.1% ± 21.5% of predicted, FEV1 117.5% ± 26.5% of predicted, and FEV1/FVC ratio 79.7% ± 4.6%. The mean bronchodilator reversibility was 2.1% ± 2.8% (SD). Chronic airflow limitation, defined as the ratio of forced expiratory volume in 1 s (FEV1) to the forced vital capacity (FVC), i.e., FEV1/FVC < 0.7 and FEV1 < 80% of the predicted value, and bronchial reversibility in response to bronchodilators were not observed. Bronchial responsiveness to methacholine was heightened in one patient. Cough reflex sensitivity, as assessed by estimation of the capsaicin cough threshold, was increased in 3 patients (Table 1).

**Table 1:** Characteristics of the 10 patients with CIC

**Case**	**1**	**2**	**3**	**4**	**5**	**6**	**7**	**8**	**9**	**10**
Age (yr)	63	70	68	69	67	55	62	71	47	63
Gender	M	F	M	F	F	M	F	F	F	F
Smoking	ex-smoker	never	never	never	never	never	never	never	never	never
Duration (Month)	49	68	66	70	44	64	48	27	41	62
WBC	4350	5800	8100	5600	5900	6600	4000	4900	3000	5400
Eo (%)	2.4	5.2	1.1	1.7	1.1	0	1.4	1	3.2	1.7
IgE (IU/ml)	179	20	97	119	734	75.5	16	38	20	29
FVC	2.78(81.0)	2.64(118.9)	4.67(137.0)	2.46(108.4)	3.46(149.8)	3.70(98.9)	2.94(126.2)	2.69(123.4)	2.95(110.5)	2.17(87.1)
FEV1	2.13(80.1)	1.90(118.7)	3.62(138.2)	1.93(114.9)	2.69(152.0)	3.07(96.5)	2.56(143.0)	2.20(144.7)	2.53(106.3)	1.67(80.7)
FEV1/FVC (%)	76.6	72	77.5	78.5	77.7	83	87.1	81.8	85.8	77
Reversibility (%)	1.2	2.6	−3.6	5.7	3.7	1.3	0.4	0.9	5.9	2.4
Rt-Meth (mg/mL)	20000<	20000<	20000<	20000<	20000<	20000<	5000	20000<	20000<	20000<
Cough (μM)	0.98	31.3	62.5	31.3	1.95	15.6	3.91	0.49	0.49	1.95

Eo: Eosinophil. FEV1: forced expiratory volume in 1 s. FEV1%: the ratio of forced expiratory volume in is (FEV1) to forced vital capacity (FVC). RT-Meth: respiratory threshold of methacholine (mg/ml) required to cause a 20% or more fall in FEV1 from the baseline value. Cough: The capsaicin cough threshold (C5) was defined as the lowest concentration of inhaled capsaicin eliciting five or more coughs.

Positive results of sputum culture for BM, *Aspergillus fumigatus*, *Aspergillus niger*, and *Candida* were detected in 7, 3, 3, and 2 patients, respectively.

The allergological findings of the 10 patients are summarized in Table 2. Eosinophilia in the induced sputum was observed in 1 patient. Although no immediate cutaneous reactions to *Aspergillus* or *Alternaria* were observed, positive results for immediate cutaneous reaction to *Candida*, *B. adusta*, and *S. commune* were observed in 4, 6, and 1 patient, respectively. In addition, positive results for late cutaneous reaction to *Candida*, *B. adusta*, and *S. commune* were observed in 1, 5, and 1 patient, respectively. Specific IgE for house-dust and mites were positive only in Patient No. 1. Positive ratios on LST against *B. adusta* were observed in 6 patients.

**Table 2:** Allergological findings of the 10 patients with CIC

**Case**		**1**	**2**	**3**	**4**	**5**	**6**	**7**	**8**	**9**	**10**
Fungal culture		BM, An	Can, Af, BM	Can	BM	BM	none	Af, BM	BM, An	BM	Af, An
Eo. In sputum		0%	5%	1%	0%	0%	0%	0%	0%	1%	0%
**Skin tests**											
*Aspergillus*	imme	4x4	3x3	2x2/0x0	0x0/0x0	5x6	2x2/0x0	0x0/0x0	2x2/0x0	4x4	4x4
	late	4x4	4x4	2x3		5x6	3x3	0x0/0x0	5x5	0x0	3x3
*Alternaria*	imme	2x2	3x3	4x4	0x0/0x0	2x2	3x3/0x0	0x0/0x0	0x0/3x3	5x5	ND
*Candida*	imme	5x5	10x10/22x22	0x0/0x0	12x12/16x14	9x10/40x31	4x4/0x0	8x8/28x30	0x0/3x3	ND	ND
	late	4x4	9x6	5x5		6x6	3x3/8x8	10x10	0x0/3x3		
*B.adusta*	imme	5x6/25x25	4x4/0x0	6x6/0x0	0x0/12x8	0x0/10x8	2x2/0x0	6x6/26x24	0x0/6x6	6x6/16x18	0x0/10x10
	late	5x5	4x4/10x8	9x8	7x7/10x10	15x14	7x7	15x15	10x10	4x5	4x4
*S. commune*	imme	0x0/0x0	2x2/0x0	5x5/0x0	0x0/0x0	4x4	4x4/0x0	0x0/0x0	0x0/0x0	4x4	4x9
	late	0x0/9x9	8x5	8x7	0x0/0x0	7x6	3x3	10x10	3x3	9x9	9x9
**Specific IgE**											
House-dust		1.86	0.34>	0.34>	0.34>	0.34>	0.34>	0.34>	0.34>	0.34>	0.34>
Mite		2.69	0.34>	0.34>	0.34>	0.34>	0.34>	0.34>	0.34>	0.34>	0.34>
*Aspergillus*		0.34>	0.34>	0.34>	0.34>	0.4	0.34>	0.34>	0.34>	0.34>	0.34>
*Alternaria*		0.34>	0.34>	0.34>	0.34>	0.34>	0.34>	0.34>	0.34>	0.34>	0.34>
*Cladosporium*		0.34>	0.34>	0.34>	0.34>	0.34>	0.34>	0.34>	0.34>	0.34>	0.34>
*Penicillium*		0.34>	0.34>	0.34>	0.34>	0.34>	0.34>	0.34>	0.34>	0.34>	0.34>
*Candida*		0.34>	0.34>	0.34>	0.34>	7.05	0.34>	0.34>	0.34>	0.34>	0.34>
*Trichophyton*		0.34>	0.34>	0.34>	0.34>	0.34>	0.34>	0.34>	0.34>	0.34>	0.34>
**LST**		negative	positive	positive	positive	positive	negative	positive	positive	ND	ND

Eo.= eosinophil.

BM; basidiomycetous fungi, Can; *Candida*, Af; *Aspergillus fumigatus*, An; *Aspergillus niger.*

imme; immediate phase response, late; late phase response.

LST; lymphocyte stimulation tests, ND; not done.

The efficacies of low-dose ITCZ therapy in 10 patients are summarized in Table 3. Significant changes in Δ cough scale or MID in domains of the J-LCQ were observed in 3 and 5 patients, respectively. The Δ cough scale was correlated with changes in some domains of the J-LCQ [total (r = –0.73, *P* < 0.05), psychological (r = –0.73, *P* < 0.05), and social (r = –0.71, *P* < 0.05)], but not with changes in the physical domain or in capsaicin cough threshold. There were significant differences in the changes in total score (*P* < 0.05) and in the social domain (*P* < 0.05) and Δ cough scale (*P* < 0.05) between positive and negative results of immediate skin test for *B. adusta*.

**Table 3:** Changes in domains of J-LCQ in 10 patients with CIC pre- and post-low-dose ITCZ therapy

		**pre-**	**post-**				**pre-**	**post-**				**pre-**	**post-**				**pre-**	**post-**				**pre-**	**post-**		
**Case**	**1**					**2**					**3**					**4**					**5**				
J-LCQ																									
total		14.29	15.92	1.63	*		16.13	16.61	0.48			18.63	17.61	−1.02			14.84	16.71	1.87	*		16.71	18.02	1.31	*
physical		4.75	6.13	1.38	*		4.63	5.25	0.62	*		5.38	5.5	0.12			5.13	5.5	0.37	*		5	5.5	0.5	*
social		4.25	4.5	0.25	*		5.5	5.5	0			6.25	6.25	0			5	5.5	0.5	*		6	6.5	0.5	*
psychological		5.29	5.29	0			6	5.86	−0.14			7	5.86	−1.14			4.71	5.71	1	*		5.71	6.02	0.31	
Cough VAS (mm)		30	26	−4	*		22	24	2			17	32	15			10	8	−2			74	58	−16	*
Cough No.		3	4	1			9	7	−2			7	8	1			3	3	0			6	6	0	
**Case**	**6**					**7**					**8**					**9**					**10**				
J-LCQ																									
total		18.92	16.81	−2.11			17.95	17.52	−0.43			19.59	20.5	0.91			8.32	12.96	4.64	*		16.2	20.63	4.43	*
physical		6.13	5.63	−0.5			5.63	5.38	−0.25			6.38	6.5	0.12			3.25	4	0.75	*		5.13	6.88	1.75	*
social		6.5	5.75	−0.75			5.75	6	0.25	*		6.5	7	0.5	*		2.5	4.25	1.75	*		5.5	6.75	1.25	*
psychological		6.29	5.43	−0.86			6.57	6.14	−0.43			6.71	7	0.29			2.57	4.71	2.14	*		5.57	7	1.43	*
Cough VAS (mm)		39	44	5			34	26	−8			18	30	12			52	31	−21	*		61	43	−18	*
Cough No.		7	7	0			4	4	0			2	9	7	*		3	4	1			5	4	−1	

The mean MID of the J-LCQ corresponding to a small change in the four GRCQ score was 1.3. The MIDs for domains were as follows: physical 0.2, social 0.2, and psychological 0.8. A change in △ cough scale by more than 15 mm was taken to be significant. * Significant improvement in each MID of J-LCQ, Cough VAS after the low dose ITCZ therapy.

In this study, although the efficacy of ITCZ therapy against SMIT was not estimated quantitatively, it was remarkable that such complaints almost disappeared. Positive results for improvement of this SMIT were observed in 6 patients in the BM colonization-positive group (85.7%) and none in the BM colonization-negative group (0%). There was a significant difference in the positive ratio for improvement of this cough-related laryngeal sensation between the two groups.

There were no significant differences in the change in total score or in the domains of LCQ and Δ cough scale between groups with positive and negative results for sputum culture of BM fungi.

## Discussion

By focusing on the role of fungal colonization in sensitization of patients with allergic fungal cough [9], it has recently been demonstrated that colonization by *B. adusta* is necessary in the process of sensitization to this fungus [27]. Although the routine use of antifungal drugs against fungus-sensitized asthma or severe asthma with fungus sensitization [28] requires further evaluation, in such cases of FACC or AFC, antifungal therapy is expected to have advantages for reducing or eradicating the colonizing antigen and thus preventing the sensitization process [9-12,29].

Although the presence of BM fungi in induced sputum has been reported to be an important clinical finding associated with CIC [13], at least three types of association of BM fungi with CIC are possible: 1) sole colonization with BM fungi; 2) sensitization with BM fungi; and 3) colonization and/or sensitization by BM fungi in addition to established CIC. It will be important to perform prospective studies to evaluate both the appropriate dose and period for performing ITCZ therapy in a larger number of CIC patients in the near future. Therefore, it does not seem to be a simple matter to appropriately evaluate the efficacy of antifungal therapy for CIC. A number of problems remain; what should the target of anti-fungal therapy be? How should the efficacy of the proposed antifungal drug be evaluated? The goal of cough therapy is generally considered to be complete remission of cough symptoms. However, even if impossible, it may be a well controlled-state with improvement of cough threshold, cough severity, cough frequency, or quality of life.

In this study, we investigated some important clinical signs [12] or allergological findings to predict the efficacy of antifungal therapy and to clarify some problems raised in the process of evaluating the clinical efficacy of the proposed antifungal drug, especially with regard to both colonization by BM fungi and sensitization to *B. adusta*[27]. The positive ratio of BM cultured from the sputa of 7 CIC patients (70.0%) in the present study was as high as that reported previously (62.5%) [13]. This result reconfirmed the importance of the presence of BM fungi in induced sputum of CIC. The positive high ratio of both skin reaction and LST against *B. adusta* suggested significant correlations with sensitization to this fungus and CIC patients.

*B. adusta* causes “Yakeirotake cough” [11], and has attracted attention because of its potential role in increasing the severity of cough symptoms in FACC patients by sensitization to this fungus. Sautour et al. reported that in outdoor samples, *B. adusta* (8%) was the third most frequent species, especially in summer, and was the third and fourth most common species in the adult hematology unit (13%) and the pediatric hematology unit (11%), respectively, at a French hospital [30]. They also mentioned that the concentration of this fungus was particularly high during the winter 2006/07, with a percentage close to 30% in indoor samples. *B. adusta* is a wood decay BM fungus with a worldwide distribution [31] and therefore this fungal antigen may be a matter of clinical concern.

Based on the mean MID [26] of the J-LCQ [25], the efficacy of low-dose ITCZ therapy was demonstrated in 5 patients. Although the MID may not always reflect sufficient improvement of patient’s cough symptoms, it is true that there were significant changes in Cough VAS in 3 of the 5 patients. Therefore, these results do not exclude the possibility of future research on the efficacy of ITCZ therapy in CIC patients. It is also remarkable that changes in Cough VAS were correlated with changes in domains in the J-LCQ.

Despite investigating the allergological pattern with regard to fungal sensitization, none of the allergological findings except positive results for immediate cutaneous reaction against *B. adusta* demonstrated significant correlations with changes in cough VAS or J-LCQ. Appropriate allergological findings, from the aspect of sensitization of *B. adusta*, may lead to success in predicting the efficacy of antifungal therapy in CIC patients.

Although the clinical manifestation, which is represented as a sensation of mucus in the throat [12], has been believed to be an important cough-related laryngeal sensation, it was shown to be correlated with colonization by BM fungi. This small but important symptom may have been overlooked because the symptom is not picked up or reflected even with use of capsaicin cough tests, cough VAS, or LCQ; nevertheless, this symptom and cough itself should also be treated.

## Conclusions

At present, it is not possible to conclude whether ITCZ therapy provides sufficient relief in CIC patients. However, this study suggested both the possible applicability of low-dose ITCZ therapy for treatment of CIC patients with regard to BM allergy and the necessity of development of a new assessment questionnaire for cough-related laryngeal sensations.

### Consent

Written informed consent was obtained from the patient for publication of this Case report and any accompanying images. A copy of the written consent is available for review by the Editor-in-Chief of this journal.

## Abbreviations

(CIC): Chronic idiopathic cough; (AFC): Allergic fungal cough; (FACC): Fungus-associated chronic cough; (BM): Basidiomycetous; (ITCZ): Itraconazole; (SDA): Sabouraud’s dextrose agar; (LST): Lymphocyte stimulation test; (VAS): Visual analog scale; (LCQ): Leicester Cough Questionnaire; (J-LCQ): Japanese version of LCQ; (MID): Minimal important difference; (GRCQ): Global Rating of Change Questionnaires; (FEV1): The ratio of forced expiratory volume in 1 s; (FVC): The forced vital capacity.

## Competing interests

All authors declare that they have no competing interests that might be perceived to influence the results and discussion reported in the present manuscript.

## Authors’ contributions

Dr. Ogawa, Dr. Takeuchi and Dr. Makimura all belong to Fungus association cough research society. Especially Dr. Takeuchi and Dr. Makimura contribute to identifying fungi. Dr.Fujimura is general conductor of this study. All authors read and approved the final manuscript.
